# Activation and overexpression of the aryl hydrocarbon receptor contribute to cutaneous squamous cell carcinomas: an immunohistochemical study

**DOI:** 10.1186/s13000-018-0740-x

**Published:** 2018-08-25

**Authors:** Zhan-Yan Pan, Jia Chen, Qiong Wu, Ting-Ting Hu, Lingyi Lu, Qiang Ju

**Affiliations:** 1 Department of Dermatology, Renji Hospital, School of Medicine, Shanghai Jiaotong University, Pujian Road 160, Shanghai, 200127 China; 2 Shanghai Skin Diseases Hospital, Affiliated to Tongji University, Shanghai, China; 3 Department of Dermatology, Ningbo First Hospital, Ningbo, Zhejiang, China

**Keywords:** Aryl hydrocarbon receptor, Nonmelanoma skin cancer, Immunohistochemical study

## Abstract

**Background:**

In vitro studies showed that the aryl hydrocarbon receptor (AHR) contributed to the development of cutaneous squamous cell carcinomas, but supporting clinical data are lacking.

**Methods:**

Immunohistochemical analysis was used to detect the expression of AHR, CYP1A1, EGFR, and Ki-67 in 10 actinic keratosis (AK) cases, 10 Bowen disease (BD) cases, 20 cutaneous squamous cell carcinoma (cSCC) cases and 20 normal skin samples. H-scores were used to assess the immunoreactivity.

**Results:**

Weak positive AHR immunoreactivity was found in all normal skin samples, while strong positive AHR immunoreactivity was found in atypical squamous proliferation (AK, BD and cSCC) cases. H-scores and the rate of strong immunostaining of the atypical squamous proliferation cases were higher than those of normal controls (*p* < 0.01). Nuclear expression of AHR was higher in atypical squamous proliferation cases than in normal controls (*p* < 0.01). H-scores and the nuclear expression rate of AHR were significantly higher in AK and BD cases than cSCC cases (*p* < 0.01). CYP1A1 expression was low and showed no differences among the four studied groups (*p* > 0.05). The H-score of AHR was positively correlated with EGFR expression (*r* = 0.54, *p* < 0.01) in atypical squamous proliferation cases but was not correlated with CYP1A1 (*r* = − 0.17, *p* = 0.295) and Ki-67 (*r* = − 0.48, *p* = 0.222) expression.

**Conclusion:**

AHR plays a vital role in cSCC pathogenesis. The overexpression and activation of AHR are involved in the early development of skin cancers. AHR expression correlates with EGFR expression and may influence cell proliferation. AHR is a valuable therapeutic target for skin cancers.

## Background

Nonmelanoma skin cancer (NMSC) is the most common type of carcinoma, accounting for at least 40% of cancer cases [1]. Although the mortality rate caused by NMSC has decreased in the last 30 years, the incidence of this disease has increased [2]. The prevalence of skin cancer is higher than that of breast cancer and all other cancers [3]. This disease is an enormous economic burden on the medical system.

Environmental factors, such as ultraviolet radiation and environmental pollution, contribute to skin cancer [4]. Epidemiological studies showed that most skin cancers resulted from solar and ultraviolet radiation exposure. Many reports have confirmed the role of polycyclic aromatic hydrocarbons (PAHs) and dioxins in the development of cSCC [4]. Multiple studies focused on the molecular mechanisms of these environmental factors in the occurrence of cSCC. Various molecular markers, including p53 [5], nuclear factor-kappa B, the activator protein-1 complex [6] and human epidermal growth factor receptor (EGFR) [7], are activated by the environmental factors and contribute to the development of cSCC. However, how environmental factors activate these molecules is not clear so far [8].

The aryl hydrocarbon receptor (AHR) is a ligand-activated transcription factor from the basic-helix-loop-helix (bHLH)/PER-ARNT-SIM homology region (PAS) family. AHR is detected in many human tissue extracts, including lung, liver, thymus, kidney, and skin. AHR residing in the cytoplasm can be activated by environmental factors and translocate into the nuclei of in vitro cultured skin cells [9]. Epidemiological studies confirmed correlations between skin cancer and exposure to AHR ligands in toxic environmental pollutants (such as PAHs). An animal study showed that AHR was essential for skin tumor induction by benzo[a]pyrene [10]. Moreover, UVB irradiation can activate the AHR pathway, and UVB-induced COX-2 gene expression is AHR-dependent [11]. These results hinted that the AHR pathway is involved in the development of skin cancers and might serve as a bridge between environmental factors and oncogenes.

Although these laboratory studies indicated that AHR might play a role in the pathogenesis of skin cancers, to the best of our knowledge, no clinical data have confirmed these results. This study aimed to evaluate the role of AHR and its downstream gene CYP1A1 in cSCC pathogenesis by examining its immunohistochemical expression in skin biopsies of normal controls and actinic keratosis (AK), Bowen disease (BD) and cutaneous squamous cell carcinoma (cSCC) patients and correlating their expression levels with the cell proliferation markers EGFR and Ki-67.

## Methods

### Study population

This retrospective study was carried out on 60 patients, including 40 cases with atypical squamous proliferation (10 cases with AK, 10 cases with BD and 20 cases with cSCC) and 20 normal controls. These patients were treated at Shanghai Skin Diseases Hospital or Ren Ji Hospital between 2011 and 2015. We collected the paraffin blocks from the archives of the pathology departments in the two hospitals. Twenty normal skin paraffin blocks were taken from patients undergoing plastic surgery. All the samples in this study were taken from the sun-exposed sits (head and neck) to eliminate the difference induced by UV-exposure. Clinical data of the cases were shown in Table 1.This study was conducted according to the Declaration of Helsinki Principles and was approved by the institutional review board at Renji Hospital.

**Table 1:** Clinical data of studied cases

Variable	Normal skin		AK		BD		cSCC	
	*N* = 20		*N* = 10		*N* = 10		*N* = 20	
	No.	%	No.	%	No.	%	No.	%
Age	64.6 ± 10.33		68.8 ± 9.08		66.9 ± 10.42		70.5 ± 5.53	
Gender								
Male	7	35	7	70	5	50	12	60
Female	13	65	3	30	5	50	8	40
Site								
Head	15	75	10	100	10	100	17	85
Neck	5	25	0	0	0	100	3	15

### Immunohistochemical assay

The immunohistochemical analysis evaluated the expression of AHR and its downstream genes. Four-micron-thick paraffin slides were dewaxed with xylene for 30 min and then rehydrated using graded ethanol concentrations. Endogenous peroxidase was blocked with 3% hydrogen peroxide (Maixin, Fuzhou, China) for 10 min at room temperature. For the antigen retrieval procedure, we heated the slides at 98–99 °C for 15 min in a pressure cooker. All slides were then incubated with goat serum (Maixin, Fuzhou, China) for 20 min to reduce nonspecific staining. Then, the slides were incubated at 4 °C overnight with primary antibody, such as rabbit anti-human AHR polyclonal antibody at 1:100 dilution (sc-5579; Santa Cruz Biotechnology, Shanghai, China), mouse anti-human CYP1A1 monoclonal antibody at 1:50 dilution (sc-25,304; Santa Cruz Biotechnology, Shanghai, China), rabbit anti-human EGFR monoclonal antibody (SP111; Maixin, Fuzhou, China) and rabbit anti-human Ki-67 monoclonal antibody (SP6; Maixin, Fuzhou, China). Biotinylated anti-mouse and anti-rabbit antibodies (Maixin, Fuzhou, China) were applied for 15 min in a humidified chamber at room temperature. Finally, a DAB Kit (Maixin, Fuzhou, China) was used for the final chromogen analysis. PBS was used as the negative control.

### Immunohistochemical results of the semi-quantitative analysis

According to the molecules biology, brown cytoplasmic and/or nuclear staining for AHR was considered positive; Brown cytoplasmic staining for CYP1A1 was considered positive; Brown membranous staining for EGFR and brown nuclear staining for Ki-67 was considered positive. The following data were recorded [12]: the intensity of the stain was graded as weak, moderate, or strong; the staining pattern was recorded as cytoplasmic, membranous, or nuclear; a positive percentage rate was given in tumor islands and the overlying epidermis after counting 500 cells in each section [13]. H-scores were calculated in all specimens. The equation [14] for the H-score = 1 × % of weakly stained cells+ 2 × % moderately stained cells+ 3 × % of strongly stained cells.

### Statistical analysis

SPSS software version 17.0 (SPSS Inc., Chicago, IL, USA) was used for statistical analyses. The positive rates of AHR, CYP1A1, EGFR and Ki-67 expression in the 4 studied groups were analyzed using the chi-square test and Fisher’s exact test. Mann–Whitney U-tests and Kruskal–Wallis tests were used for comparison between quantitative variables (H-score). Spearman’s correlation was used to measure the linear association. Differences were considered significant when a *p* < 0.05 was obtained.

## Results

### Immunohistochemical expression of AHR and CYP1A1 in the studied groups

#### Normal skin

**Fig. 1:**
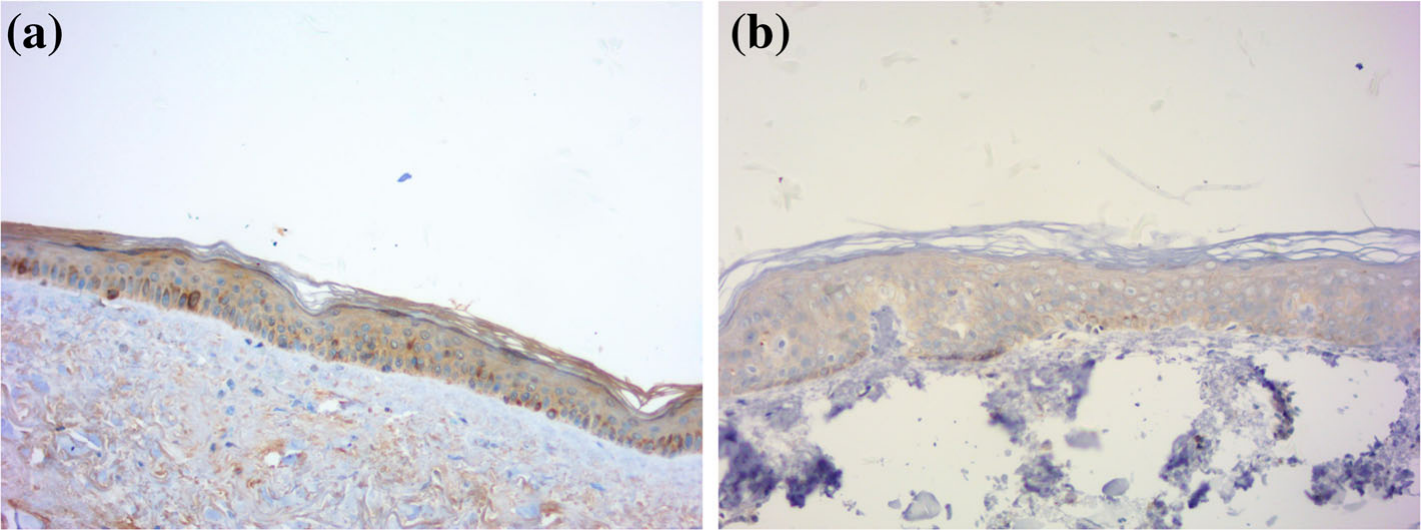
AHR and CYP1A1 in normal skin. **a** Weak positive AHR immunoreactivity with a cytoplasmic pattern in epidermis. **b** Weak CYP1A1 immunoreactivity with a cytoplasmic pattern in epidermis. (immunoperoxidase ×200)

Weak positive AHR immunoreactivity was observed in all sections with a cytoplasmic pattern in the epidermis. (Fig. 1, Table 2). The intensity of expression ranged from weak to moderate in the epidermis (Table 3). Positive immunoreactivity was also noted in sebocytes and sweat gland ducts. Seven (35%) sections showed weak CYP1A1 immunoreactivity with a cytoplasmic pattern in the epidermis. (Fig. 1).

**Table 2:** Expression of AHR and CYP1A1 in NMSC and normal skin

Group	n	AHR exprssion (%)	AHR(H-score)	CYP1A1(%)	CYP1A1(H-score)
Normal skin	20	20 (100)	80.9 ± 25.79	7 (35)	35.3 ± 51.07
AK	10	10 (100)	211.1 ± 44.29	4 (40)	46.9 ± 69.64
BD	10	10 (100)	205.8 ± 52.93	4 (40)	58 ± 71.85
cSCC	20	16 (80)	133.15 ± 86.67	6 (30)	39.3 ± 62.48
*p value*		>0.05	<0.01	>0.05	>0.05

**Table 3:** Expression of AHR, CYP1A1, EGFR and Ki-67 in studied groups

Variable	Normal skin		AK		BD		cSCC	
	*N* = 20		*N* = 10		*N* = 10		*N* = 20	
	No.	%	No.	%	No.	%	No.	%
H-score(AHR)	80.9 ± 25.79		211.1 ± 44.29		205.8 ± 52.93		133.15 ± 86.67	
Intensity								
weak	15	75	1	10	0	0	3	15
moderate	5	25	1	10	6	60	5	25
strong	0	0	8	80	4	40	8	40
Pattern								
cytoplasmic	17	85	10	100	10	100	16	80
nuclei	4	20	9	90	7	70	7	35
H-score(CYP1A1)	35.3 ± 51.07		46.9 ± 64.25		58 ± 76.31		39.3 ± 62.48	
H-score(EGFR)	68.1 ± 13.46		179.3 ± 43.13		237.4 ± 55.67		183.5 ± 48.46	
H-score(Ki-67)	20.4 ± 5.56		71.3 ± 62.69		174.6 ± 51.03		197.45 ± 56.32	

#### Actinic keratosis and Bowen disease

**Fig. 2:**
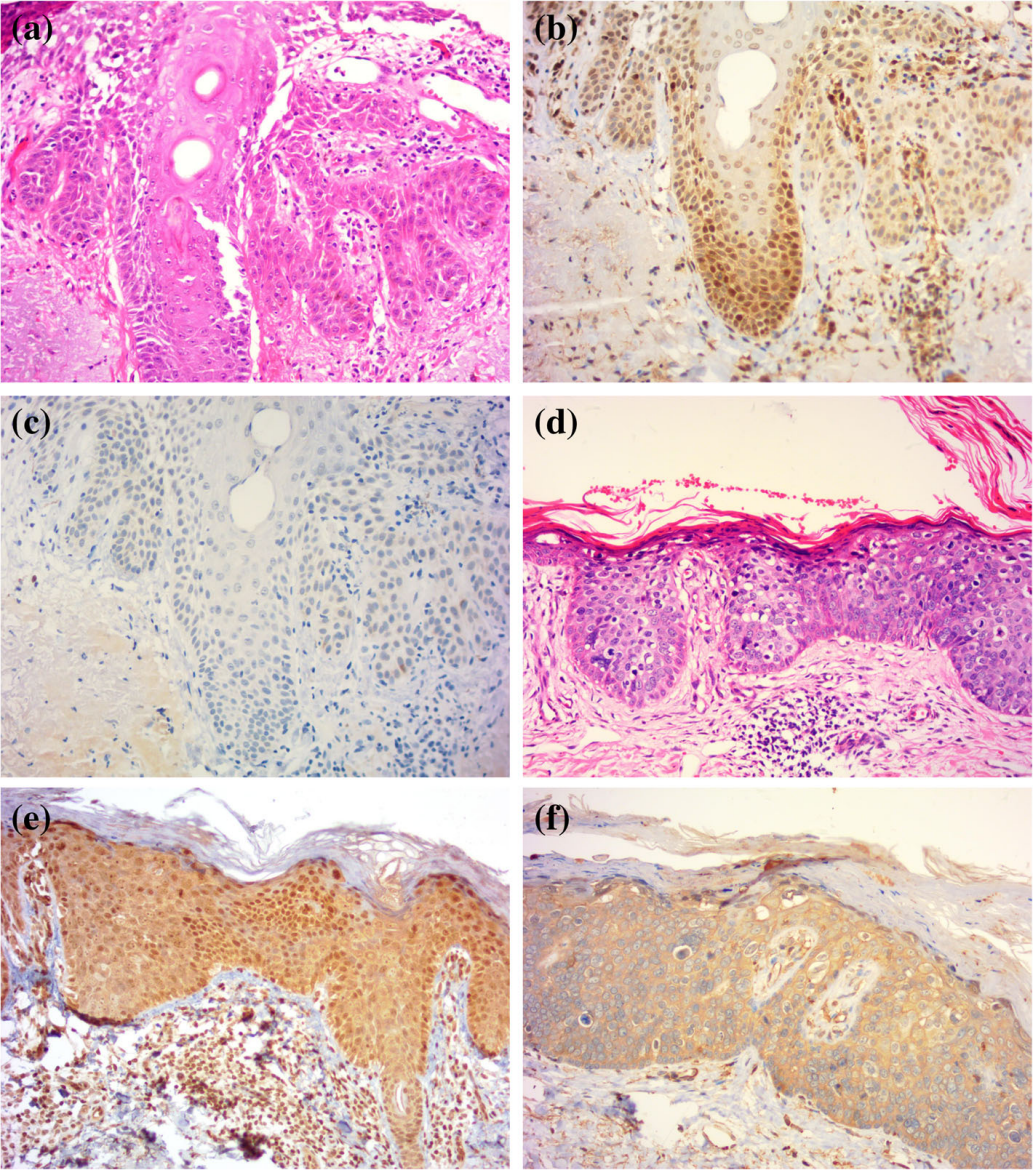
AHR and CYP1A1 in AK and BD cases. **a** Haematoxylin and eosin stain for AK. **b** Strong positive AHR immunoreactivity with a nuclear pattern in AK cells. **c** Negative CYP1A1 immunoreactivity in AK cells. **d** Haematoxylin and eosin stain for BD. **e** Strong positive AHR immunoreactivity in both the nuclei and cytoplasm in BD cells. **f** Moderate CYP1A1 immunoreactivity with a cytoplasmic pattern in BD cells. (immunoperoxidase × 200)

Strong positive AHR immunoreactivity was found in all AK cases and BD cases and showed both cytoplasmic and nuclear patterns (Fig. 2, Table 3). The intensity of expression in tumor cells ranged from moderate to strong. Positive dermal immunoreactivity was noted in inflammatory cells. Weak to moderate positive CYP1A1 immunoreactivity was found in 4 (40%) AK cases and 4 (40%) BD cases, indicating a cytoplasmic pattern.

#### Cutaneous squamous cell carcinoma

**Fig. 3:**
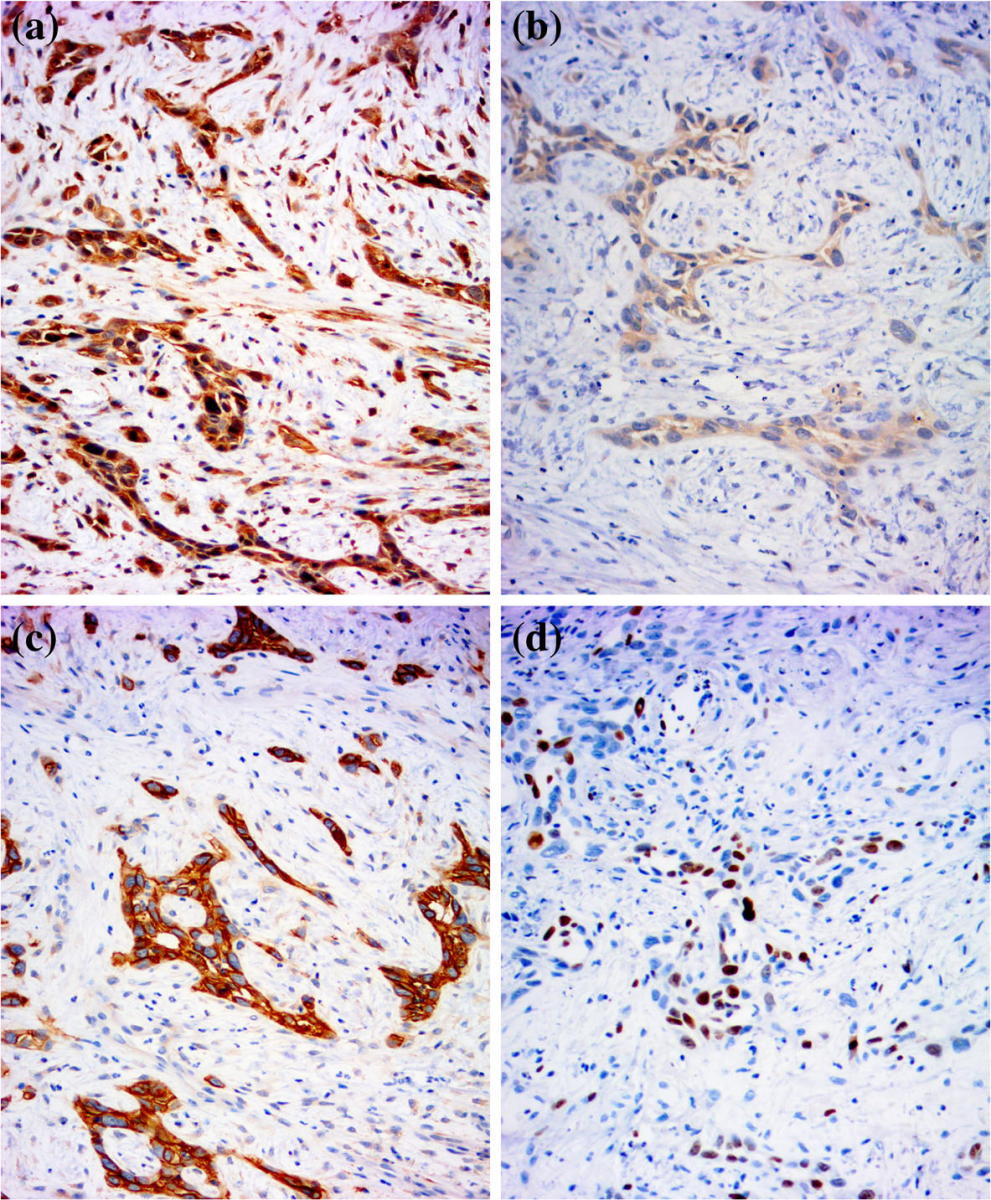
AHR, CYP1A1, EGFR, and Ki-67 in a cSCC case. **a** Strong positive AHR immunoreactivity in both the nuclei and cytoplasm in SCC cells. **b** Weak CYP1A1 immunoreactivity with a cytoplasmic pattern in SCC cells. **c** Strong positive EGFR immunoreactivity in SCC cells. **d** Moderate Ki-67 immunoreactivity in SCC cells. (immunoperoxidase × 200)

AHR was expressed in 16 (80%) cSCC cases. Positive cell nuclear immunoreactivity was noted in 7 (35%) cases. The intensity of expression ranged from weak to strong. AHR was mainly expressed in tumor islands and stromal inflammatory cells. Weak positive CYP1A1 immunoreactivity was found in 6 (30%) of the examined cases, indicating a cytoplasmic pattern. (Fig. 3).

#### Comparison between AHR and CYP1A1 expression in the groups of atypical squamous proliferation cases versus normal skin

The AHR expression percentage showed no difference (*p* > 0.05) between two groups, atypical squamous proliferation cases (AK, BD and cSCC cases) and normal controls, but the H-score value was significantly higher in atypical squamous proliferation cases than normal controls (*p* < 0.01) (Table 2). The strong AHR immunostaining rate and the number of cases with nuclear expression of AHR were significantly higher in atypical squamous proliferation cases than normal controls (*p* < 0.01) (Table 3). The expression percentage, H-score and expression pattern of CYP1A1 showed no difference between the two groups (*p* > 0.05) (Table 2).

#### Precancerous lesions (AK and BD cases) versus cSCC

A comparison of AHR expression percentage showed that there was no difference among these three groups (*p* > 0.05). H-scores were significantly higher in the AK and BD cases than the cSCC cases (*p* < 0.01). Nuclear AHR expression was also significantly higher in AK and BD cases (*p* < 0.01). For the CYP1A1 expression percentage, H-score and expression pattern, no difference was shown among the three groups (*p* > 0.05).

#### Relationship among AHR, CYP1A1 and cell proliferation markers (EGFR and Ki-67)

The H-score of AHR was positively correlated with that of EGFR (*r* = 0.54, *p* < 0.01) in the atypical squamous proliferation group. The H-score of AHR was not significantly associated with that of CYP1A1 (*r* = − 0.17, *p* = 0.295) or Ki-67 (*r* = − 0.48, *p* = 0.222) in the atypical squamous proliferation group.

## Discussion

AHR is a member of the bHLH/PAS family and widely expressed in many animals and humans. Many studies suggested that AHR was involved in various signaling pathways critical to cell proliferation and differentiation [15]. Abnormal overexpression and activation of AHR contributed to the development of many cancers [16, 17]. Although a few laboratory studies suggested that AHR played an important role in the pathogenesis of skin cancers, no clinical data have confirmed these results [18, 19].

In this study, AHR was expressed in all normal skin samples. Its expression was mainly observed in the cytoplasm of the basal and suprabasal layer cells. AHR was also observed in sebocytes and sweat gland ducts. These results were in line with the cell culture studies and suggested that constitutive expression of AHR was necessary for healthy skin cells [20].

AHR was overexpressed in cSCC and its premalignant lesions. The H-score and strong immunostaining rate were higher in atypical squamous proliferation cases than normal controls. These results confirmed that overexpression of AHR is associated with the cSCC. The AHR nuclear expression, a characteristic of AHR activation, was also higher in atypical squamous proliferation cases. Previous studies emphasized that abnormal AHR expression and activation inhibited the functional expression of anti-oncogenes and altered cell survival, proliferation, and differentiation in breast and liver cancer cells [15]. We hypothesized that both overexpression and activation of AHR contribute to the pathogenesis of cSCC. Furthermore, in this study, both the H-score and nuclear pattern of AHR were significantly higher in precancerous lesions (AK and BD cases) than cSCC cases. In vitro study showed that AHR regulated genomic integrity by affecting both, nucleotide excision and homologous recombination repair [21]. AHR served as an anti-apoptotic factor in UVB-induced keratinocytes apoptosis [22].Taken together, these findings indicate that AHR may play an important role in the early events of cancer development.

To our surprise, the expression of the AHR downstream gene CYP1A1 showed no differences in the four study groups. These results may be explained by the fact that AHR did not use the “classical” AHR/CYP1A1 pathway but induced other genes, such as CYP1B1, CYP1A2, and CYP2S1 [20, 23]. This issue should be further studied.

Recently, Fritsche et al. [11] confirmed the intracellular formation of the AHR ligand 6-formylindolo [3,2-b]carbazole (FICZ) after UVB irradiation of a human keratinocyte cell line, and AHR activation then induced EGFR internalization and activation. Another study showed that overexpression of AHR stimulated the proliferation of cancer cells [24]. Therefore, we chose two cell proliferation markers, EGFR, and Ki-67, in this study. The results showed the positive correlation between AHR and EGFR and confirmed the existence of the AHR-EGFR pathway.

## Conclusion

In conclusion, AHR plays a vital role in cSCC pathogenesis. Both overexpression and activation of AHR may participate in the early development of skin cancers. AHR expression was correlated with EGFR expression and may influence cell proliferation. These results indicate the AHR is a valuable therapeutic target for skin cancers.

## Abbreviations

AHR: Aryl hydrocarbon receptor
AK: Actinic keratosis
BD: Bowen Disease
bHLH: Basic-helix-loop-helix
cSCC: Cutaneous squamous cell carcinoma
EGFR: Epidermal growth factor receptor
NMSC: Nonmelanoma skin cancer
PAHs: Polycyclic aromatic hydrocarbons
PAS: PER-ARNT-SIM homology region
